# A thematic analysis of the subjective experiences of mothers with borderline personality disorder who completed Mother-Infant Dialectical Behaviour Therapy: a 3-year follow-up

**DOI:** 10.1186/s40479-024-00269-w

**Published:** 2024-10-28

**Authors:** Alexandra Giles, Anne Sved Williams, Stephanie Webb, Phoebe Drioli-Phillips, Amelia Winter

**Affiliations:** 1 School of Justice and Society, University of South Australia, Adelaide, SA Australia; 2 Women’s and Children’s Health Network, North Adelaide, SA Australia; 3 School of Psychology, The University of Adelaide, Adelaide, SA Australia

**Keywords:** Borderline personality disorder, Mothers, Thematic analysis, Dialectical behaviour therapy, Qualitative, Intervention

## Abstract

**Background:**

Perinatal borderline personality disorder (BPD) is a common condition in perinatal mental health settings with few specialised treatment options, and little is known about the enduring effects of available treatment programs. This study explored the follow-up experiences of women with BPD after completing Mother-Infant Dialectical Behaviour Therapy (MI-DBT).

**Methods:**

Semi-structured interviews were conducted with eight women who had completed MI-DBT 3 years prior. Reflexive Thematic Analysis was used to analyse the interviews to gain a richer understanding of these mothers’ lived experience.

**Results:**

A thematic analysis generated four main themes which indicated that participants found that MI-DBT improved their ability to hold their child in mind, be aware of their own internal state and behaviours, manage their own emotional reactions and stay calm, and manage interpersonal interactions within adult relationships. Mothers with perinatal borderline personality disorder also highlighted the need for ongoing support in the context of parenting.

**Conclusions:**

This study is the first of its kind to explore the longer-term experiences of mothers following such an intervention, giving voice to this vulnerable group of women. The findings of this study provide a greater understanding of the complex challenges experienced as part of parenting for mothers with borderline personality disorder, and provides both insight into mothers’ experiences of life after MI-DBT and the impact of the program on their lives. The clinical and research implications of the study’s findings are discussed.

**Trial registration:**

This research was retrospectively registered on 07/03/2024, ACTRN12624000225516.

**Supplementary Information:**

The online version contains supplementary material available at 10.1186/s40479-024-00269-w.

## Background

Borderline personality disorder (BPD) is a serious, pervasive, and debilitating mental illness [1], characterised by unstable interpersonal relationships, a fragile self-image, emotional instability, and impulsive behaviours [1 2]. Relationships tend to be chaotic and intense [2] due to heightened emotional reactivity, difficulty regulating intense emotional arousal [3], and increased sensitivity to cues of rejection and abandonment [4]. The accompanying impulsive and self-destructive behaviours, such as self-harm, suicide and substance use [5], make BPD an intense and disruptive illness to live with.

Often underdiagnosed, BPD affects approximately 1–6% of the general population [6], with higher rates during pregnancy and postpartum within clinical populations [7]. A systematic review and meta-analysis by Prasad and colleagues [7] revealed that the prevalence of BPD in non-clinical samples across pregnancy ranges from 7 to 27%, increasing to 10–34% among those assessed in clinical contexts such as high-risk pregnancy clinics or psychiatric settings. Postnatally, BPD rates range from 1 to 2% among non-clinical samples, to 2–35% among clinical samples. However, prevalence rates are difficult to determine because some people present clinically significant symptoms without meeting the diagnostic threshold for BPD [6], and others with BPD may function well under normal circumstances yet need additional support at times of stress. For example, BPD symptoms are often exacerbated when an individual becomes a parent [8, 9]. Despite its high prevalence, BPD is often overlooked in national reports and surveys, and is poorly researched compared to other mental health conditions [6]. Furthermore, there is considerably less awareness of BPD than of other perinatal mental health conditions [10]. This is especially concerning given the significant potential consequences for the development and functioning of an infant [11].

### The effect of BPD on mothers

Many women with BPD experience emotional vulnerability prior to becoming mothers [12], and may face increased challenges in meeting the demands of caregiving, nurturing healthy early interactions [13], and responding to their infant’s distress [12]. Many mothers with BPD report that parenting is highly stressful [14, 15], with some mothers expressing intense feelings of guilt and regret about how BPD may affect their parenting, and in turn, their children [15, 16].

Some mothers with BPD report challenges in providing stability for their family and in meeting their children’s basic needs, such as routines, housing, finances, nutrition [14], and supportive relationships [15, 16]. The intense fear of abandonment that individuals with BPD often experience can increase the likelihood of remaining in unhealthy relationships due to fear of being alone [17]. These factors and the tendency for intense and fluctuating emotions, can lead to heightened conflict and instability [18–20], which can create a stressful environment during the peri- and postnatal periods - a time already laden with emotional and physical changes [21].

Some mothers with BPD also report challenges with maintaining appropriate interpersonal boundaries with their children [14]. For example, when mothers are unable to manage their own emotions or experience, they may inadvertently look to their child to manage this for them, resulting in role-reversal or parentification of the child [14]. This can lead to further complications in the mother-child dynamic. For example, some mothers report children being hypervigilant for their mother’s safety due to witnessing their mother’s self-harm or suicide attempts [15].

Some mothers with BPD have reported difficulty maintaining a safe environment for their children, with safety concerns relating to child protection such as aggressive behaviour towards their infant, thoughts or worries about harming their child, or the child witnessing suicide attempts [14]. At times this has resulted in the removal of the child from their care [15]. While some literature suggests that individuals with BPD have limited insight into their behaviour and the effects on others [22, 23], many mothers with BPD have reported intense guilt, regret and shame regarding the impact of their behaviours on their children [15, 16], indicating some reflective functioning ability. This guilt and shame can coexist with a perception of themselves as bad parents, potentially contributing to a fractured sense of self, further eroding their self-esteem [16]. This internal struggle can increase the risk of suicidal behaviour, potentially creating a circular self-destructive pattern of behaviour [16].

Fonagy and Bateman [23] suggested that the behavioural characteristics of BPD are due to individuals having experienced dysfunctional relationships with their own parents, marked by inconsistent, insensitive and invalidating early environments. This finding is consistent with reports from mothers with BPD about their own upbringings [16]. Almost all mothers want to do the best they can for their children [24], however their behaviour is largely based on their own experiences of being parented [25]. Mothers often find some of their children’s needs harder to meet than others, and this is influenced by their own met/unmet needs as children [25, 26].

### The effect of BPD on the relationship with the baby

Completely reliant on their caregivers for their physical and psychological needs [27], the beginning of an infant’s life is a sensitive period for social, emotional and cognitive development [28]. Lacking physical means to get closer to their caregiver, newborns use other signalling behaviours such as crying (and later, other vocalisations, smiling and locomotion) to promote proximity by stimulating a response from their caregiver [27, 29]. These are referred to as attachment behaviours [30]. For mothers with BPD, displays of distress such as crying are often overwhelming [12], which can activate their own emotional dysregulation [8, 10, 31]. Ideally, when an infant’s attachment system is activated, they engage their caregiver and receive consistent responses that are sensitive to their needs [27, 32], which help them understand that their caregiver will be available and responsive in future times of need [33]. However, in some cases, mothers with BPD, who may have had similar experiences themselves, can find it challenging to maintain consistent and sensitive emotional and behavioural responses [14, 16]. This may result in mothers prioritising their own needs over those of their children [14], at times ignoring, minimising or rejecting their child’s emotions and experience [16, 34–37]. This mismatch between the child’s needs and the mother’s responses can lead to an escalation of the child’s emotional displays [3], creating a cycle of unmet needs and emotional intensity.

Over time, these patterns of inconsistent responsiveness can become entrenched [38], leaving the infant without a central figure to hold and organise their internal world [3]. With the expectation that their caregiver will be inconsistently responsive, infants can feel frightened and alone [30]. The ongoing pattern of unpredictability and insensitivity in parenting that can be found in some mothers with BPD may negatively impact attachment, increasing the risk of developing disorganised attachments [39, 40] where the child is hardwired to seek safety and connection from their caregiver but also fears them [41, 42].

### The effect of BPD on infant development

Children with disorganised attachment patterns are at higher risk of social and emotional difficulties [43] and later psychopathology [11, 44, 45]. When children experience emotional abandonment, they may struggle to recognise, understand and label their own emotions and those of others [3, 23] and subsequently fail to acquire the necessary skills to manage stressful situations and the evoked emotions [34, 46]. This can significantly impact a developing child’s emotional and behavioural self-regulatory abilities [38, 45, 46].

Repeated invalidation can lead to the child not considering their emotions valid or real [3], leading to an unstable sense of self [23] where the child distrusts their internal experience [3]. The interaction between mother and infant establishes norms for relating to others [38], so the experience of being misunderstood, dismissed, or provoking hostility can also hinder the development of a child’s interpersonal skills [46]. Research on maternal BPD has shown that in many cases, children of mothers with BPD have long-lasting difficulty maintaining healthy relationships [38, 46, 47]. It has been suggested that the interpersonal and emotional dysregulation difficulties of the caregiver are absorbed by the infant [39]; that is, children of mothers with BPD often mirror their caregiver’s distress, dependence on others, and fear of abandonment and rejection [48], which can make relationships with them intense and at times overwhelming.

Unsurprisingly, the early experiences of those with BPD often parallel the experiences of their children [15]. Even with the best of intentions, mothers with BPD, who feel needy themselves, often have difficulty meeting their infant’s needs [16] and are at greater risk of repeating the patterns of their own upbringing with their own children [24, 25, 49]. Given these challenges for mothers with BPD and the potential pervasive effects if left untreated [49, 50] early intervention is essential to changing the trajectory for both mothers and children [11, 34, 51].

### Impact of interventions

Currently, there are limited treatment options for mothers with BPD, and even fewer that specifically address the unique challenges of parenting [49]. Several programs have been developed to fill this gap, each with varying degrees of evaluation and success.

The Parenting Skills for Mothers with BPD program [52], a 12-week group training that combines cognitive-behavioural principles with DBT techniques, has shown promise in reducing parenting stress and improving mother-child interactions, although initial evaluations primarily focused on participant satisfaction rather than clinical outcomes [52]. Similarly, Coming up for Air, a four-session intervention, provides information on parenting and BPD but has so far only been evaluated for clinician acceptability rather than its effectiveness in improving parent-child relationships [49]. Serrano et al. (2023) describe various mother-infant psychotherapeutic approaches, such as “Watch, Wait, and Wonder” therapy, which aim to enhance maternal sensitivity and support healthy mother-infant interactions [53]. Rogers (2016) evaluated the Mindfulness-Based Parenting Group Intervention for Mothers with BPD (MPG-BPD), which showed improvements in maternal reflective functioning and reduced parenting stress six months post-intervention [54]. While these therapies demonstrate short-term benefits, their long-term effectiveness remains unclear, as many programs lack follow-up data to assess sustained impact on maternal mental health and child outcomes.

The Perinatal Emotional Skills Groups (ESGs), currently being evaluated in a feasibility randomised controlled trial, offer a condensed, DBT-based intervention specifically tailored to the perinatal period [55]. Early findings suggest that ESGs may be a cost-effective way to deliver essential DBT skills to a broader population of mothers with BPD, though comprehensive results are still pending.

Hellberg and colleagues (2023) underscored the need for more parenting-specific support in their systematic review of perinatal Dialectical Behaviour Therapy (DBT) interventions [56] [10, 57, 58]. While standard BPD-specific interventions like DBT, focus on treating the underlying disorder [59] by teaching participants skills aimed at assisting with their cognitive, behavioural, emotional and interpersonal difficulties [3, 60], they often do not incorporate the unique challenges of parenting. Research highlights the need for interventions that take into account the unique challenges of parenting with BPD [51] and focus on parenting and the mother-infant relationship [15, 49, 61]. However, mothers with BPD have reported difficulty finding interventions that target both their needs and those of their infants [14].

One of the most promising interventions identified is Mother-Infant Dialectical Behaviour Therapy (MI-DBT) [10, 57, 58], which stands out for its structured approach, combining traditional DBT techniques with practical mother-infant interaction exercises, directly addressing core BPD symptoms while fostering a healthier mother-infant relationship [10, 57]. Over 24 weeks, mothers with BPD learn together in a group format how to manage the challenges of parenting, learn DBT skills such as distress tolerance, emotional regulation and relationship management, tailored to support both their own mental health and their infant’s development. Quantitative data collected over multiple timepoints have shown improvements in maternal mood, mental health, reflective functioning and confidence in parenting [10, 57]. However, while initial results indicated improvement in the mother-infant relationship [10], a subsequent evaluation of the program did not [57]. Sved Williams et al., (2021) suggested, rather than reflecting anything within the dyadic relationship, the inconsistency in findings may be due to differences in observational tools used to measure the mother-infant relationship (the Care Index vs. the NCAST), or the developmental changes in children between assessments [57]. Furthermore, while a subsequent study measured child outcomes such as social and emotional development [57], results were not statistically significant, suggesting that additional support, such as individual mother-infant therapy, may be necessary [57].

To gain a deeper understanding of these inconsistencies and explore participant experiences more thoroughly, qualitative research was conducted [62]. Participants reported perceived improvements in maternal reflective functioning, emotional regulation, and quality of their relationship with their child. However, while the initial findings are promising, it remains unclear how sustainable these improvements are over time.

Despite the growing awareness of the need for parenting-specific BPD interventions [47, 49, 63, 64] research in this area is still in its infancy. Long-term follow-up studies are crucial to understanding whether the benefits observed immediately after the MI-DBT program persist as children grow and family dynamics evolve. Such studies would provide invaluable insights into the lasting impact of the intervention [65, 66] and help refine future iterations of the program to ensure enduring outcomes for both mothers and their children [4, 49]. To our knowledge, no studies have explored the long-term subjective experiences of mothers with BPD who have completed parenting-specific BPD interventions. This study aims to fill this gap by examining participant outcomes beyond the 12-month mark, offering a comprehensive evaluation of the MI-DBT program’s effectiveness over time and informing future program development.

### Research questions

1. What are the experiences of mothers 3 years after completing the MI-DBT program?;
2. What are the perceived long-term impacts of the MI-DBT program?; and.
3. What improvements could be made to the MI-DBT program or subsequent mental health support based on participant’ experiences?

## Methods

### Sample and recruitment

To participate in the MI-DBT program, mothers had to be at least 16 years old, and have at least one child under the age of 3 at the time of enrolment^1^. Mothers were either diagnosed with BPD or displayed BPD traits of severe emotional dysregulation as defined by their treating clinician, and were referred by a clinician concerned about the quality of the mother-infant relationship. Participants were excluded from participation if they did not speak English, had insufficient cognitive capacity to understand the concepts being discussed, or had significant substance abuse problems that could impact their engagement with the program.

Participants included 8 mothers who completed the MI-DBT intervention 3 years previously (Groups 1, 5 and 6) between late 2018 and mid-2019. 29 women were eligible for follow-up and were contacted by telephone. Of these women, eight women were successfully reached and agreed to participate in an interview. The remaining 21 women either had disconnected phone numbers, did not respond to phone calls or text messages, or missed their scheduled interviews after agreeing to participate. Although there are no widely agreed upon criteria for determining participant sample sizes for thematic analysis [67], a range of 6–10 interviews is often considered sufficient for a smaller project to generate rich data without overwhelming the analysis [68].

Ethics approval to conduct evaluations of the program was granted by the Women’s and Children’s Health Network Human Research Ethics Committee (HREC/13/WCHN/111). Ethics approval was obtained from both the WCHN HREC and the University of South Australia Research Ethics Committee, allowing the primary researcher to access archival interview audio recordings and transcripts.

### Data collection

At the beginning of the MI-DBT program each participant is provided with an information sheet and consent form to participate in the research component of the program. This information advises participants that the research is longitudinal, and that they may be contacted for further follow-up. Participants are advised that they may withdraw consent (verbally or by writing) for the research at any time, and that withdrawal from the evaluation component will not impact their participation in the MI-DBT intervention, or access to any future care. Participants are also asked for verbal consent to participate in each post-program interview.

The intervention protocol can be found in Appendix A. Data collection (both quantitative and qualitative) for the MI-DBT program occurred at 4 time-points: immediately prior (T1) and post (T2) participation, and at 12-months (T3) and 36-months (T4) post-intervention. The present study focuses on the T4 interview data. Two female Research Officers employed by the Women’s and Children’s Health Network (WCHN) both psychology-trained PhD candidates (PD-P and AW), conducted semi-structured interviews with eight participants^2^ between January and August 2022. Due to COVID-19 restrictions, all interviews, except one home visit, were conducted via telephone. The interviews, which varied in length from 10 to 31 min were audio recorded with participant consent, and were transcribed and analysed by the primary researcher. The research officers were not involved in delivering the MI-DBT program, and had no biases that could affect the interviews. Data analysis was undertaken by the primary researcher (AG), who was a post-graduate psychology student at the time, and had no direct contact with the study participants. AG was supervised by both academic (SW) and clinical (AESW) supervisors.

### Measures

The semi-structured interviews used open and non-judgemental questions as prompts to explore participants’ perceptions of and experiences with the program (see Appendix B for interview schedule). These structured interview questions served as prompts for the interviewers, who then followed up on women’s responses. The conversational tone allowed for flexibility, encouraging participants to share their thoughts freely. Topics covered included emotional responses, relationships (with others and infant), infant behaviour, skills learned and currently used, current supports, and suggestions/feedback regarding program improvement.

### Approach to analysis

The audio recordings of the interviews were transcribed verbatim by the primary researcher and de-identified to protect participants’ confidentiality. The participants did not subsequently see their interview transcripts. These transcripts were analysed using an inductive/reflexive thematic analysis approach [69, 70], providing a rich and detailed exploration of participants’ lived experiences following completion of MI-DBT. Thematic analysis continued with the primary researcher reading and re-reading the transcripts, noting potential areas of interest to gain familiarity with them using the comments function in Word (Phase 1: Familiarising yourself with the data). The transcripts were coded manually by searching for similar content throughout the interviews (Phase 2: Generating initial codes), occurring systematically, giving equal attention to all data items, and looking for anything that could form the basis for the developed themes. These codes were grouped into categories, and analysed for themes and sub-themes which were generated by the primary researcher (Phase 3: Searching for themes). This set of initial themes and sub-themes was reviewed and refined by reading the extracts that formed part of the theme to ensure that they fit within that theme (e.g., for a pattern). Then each theme was reviewed to ensure that it fit within the overall data set (Phase 4: Reviewing themes). At this point, each theme was refined further and a detailed analysis of each theme was written, considering how each theme fit within the overall data set, as well as the study (Phase 5: Defining and naming themes). Participant quotes illustrate each of these themes. Square brackets replace identifying information, pseudonyms are provided for anonymity, and ellipses represent speech omissions of superfluous content. In Phase 6 (Producing the report) these themes were explored further and interpreted in relation to available theories and literature.

Consolidated criteria for reporting qualitative research (COREQ) guidelines for data reporting were followed. A completed COREQ checklist is provided in Supplement 1.

### Reflexivity and positionality statement

The research team consisted of three postgraduate psychology students, a psychologist, a clinical psychologist, and a perinatal and infant psychiatrist – all of whom were women and mothers, one of which was also a grandmother. Three members had lived experience with perinatal anxiety and/or depression, and three had professional experience in maternal and infant mental health. This familiarity enriched our engagement with the data but also introduced the potential for bias in interpreting the intervention’s effectiveness and participants’ experiences. To mitigate this, we engaged in ongoing reflection and consultation with one another to ensure that our interpretations were firmly rooted in the data rather than shaped solely by personal perspectives.

## Results

To explore participants’ experiences 3 years after completion of the MI-DBT program, eight women completed interviews, from which four themes were constructed: (1) Keeping my kids in mind, (2) Awareness of self, (3) Negotiating relationships, and (4) We want more!

### Keeping my kids in mind

This theme reflects participants’ improved ability to remain aware of their children’s needs, prioritise them in their parenting, and take their perspectives into account. Mothers highlighted how the skills learned in MI-DBT helped them develop empathy, allowing them to look beyond their own emotional struggles and focus on their children, helping them better understand and respond to their children’s needs more effectively. For example, one mother described how her shift in perspective, from herself and her own emotions to those of her child, marked an important change in her parenting approach.

I think the skills certainly made a big difference that I learnt and just I suppose even just things about myself and how to sort of put that in practice with my kids as well. It’s certainly helped me, I suppose look at things a bit from their perspective as well, not just sort of what I was doing and how I was feeling (Participant 4).

Mothers also described how MI-DBT provided them with tools to manage their own emotional responses, allowing them to respond with more thoughtfulness and less impulsivity. This emotional regulation supported their ability to engage in perspective-taking, and facilitated a more balanced approach to interacting with their children, allowing them to focus more on their children’s needs. One mother, for instance, spoke about how these skills helped her better manage interactions with her autistic children.

[Susie], she’s autistic and she’s got an intellectual disability, [Peter’s] also been diagnosed as autistic as well but so it’s a bit different when I’m engaging with them I guess and like, you know using methods and skills and stuff to what I would with maybe some other kids or some like my nieces and nephews and that but it definitely has helped me to be able to, you know, calm down because their meltdowns are not easy to manage (Participant 5).

In recognising their children’s individual needs, many mothers began to recognise their important role as mothers in supporting their children’s emotional development by taking an active, rather than passive, approach to parenting, particularly during challenging transitions. For example, one mother reflected on how she worked to create a more stable and secure environment for her child after her separation from his father, demonstrating a reflective awareness of her child’s emotional needs

I think I was more aware of, sort of trying to create that sort of more sense of security for him, especially when I, you know, broke up with his father (Participant 2).

Mothers also expressed a growing desire to better understand their children’s needs and behaviours, particularly as some had since been diagnosed with, or were waiting assessment and support for, neurodevelopmental differences. This reflective engagement demonstrated a commitment to actively supporting their children’s well-being by anticipating and addressing their unique challenges. One mother spoke about actively seeking to understand her daughter’s needs and planning ahead to ensure her support as she grows older.

I’m pretty sure [Jackie] has like some sensory issues and I’m just starting the process now to get her assessed…she’s growing up really a lot and I really want to try and get some kind of, I just, like, an understanding with what we need to do to help her before she becomes a full-on teenager and it gets even harder. (Participant 1)

These new behaviours were linked to mothers’ improved skills and ability to manage their own emotions, which allowed them to focus more effectively on their role as parents, including both the joys and challenges of motherhood.

### Awareness of self

This theme relates to mothers’ improved ability to be aware of and reflect on their own internal states, emotions and behaviours. Through participation in MI-DBT, mothers described a greater capacity to recognise their emotional triggers and patterns, which allowed them to make more thoughtful and conscious choices in how they responded to emotional situations. Previously, when distressed, mothers often reacted automatically, relying on coping mechanisms that were more reactive and less intentional, such as avoidance, distraction, or hostile and aggressive behaviour.

I’d either start getting like really overwhelmed and start snapping or yelling, or I’d just distract to like no end a lot of the time. I did get free child care back then, so a lot of the time when the kids were at child care, I’d just stay in bed or I’d do like craft or whatever instead of actually kind of building my life up (Participant 7).

In addition to managing their children’s needs, mothers reflected on the guilt they felt when they couldn’t meet everyone’s needs equally. Some spoke about how their personal circumstances, such as employment or having additional children, contributed to feelings of inadequacy.

I am working now so I don’t get to spend as much time with them as I would like. (Participant 1)

When they’re having trouble during the day, it definitely does make it hard and if one wants a cuddle, they all seem to want a cuddle at the same time but I’ve got 4 kids and it’s definitely hard…It’s not easy at times, and then I just feel guilty, like I shouldn’t have had so many kids (Participant 3).

While these feelings of guilt were difficult, they also served as motivators for change. One mother reflected on how her improved emotional regulation influenced her interactions with her children.

I do whatever I need to stop it reaching that worst case scenario. You know I’ve never done anything to my girls but there are times there are times where I have reacted to or yelled in such a way where I do feel really guilty afterwards (Participant 4).

This awareness of their emotional states prompted a desire for change, leading mothers to adopt more adaptive strategies when they began to notice heightened emotions. With greater self-compassion, they reported feeling more confident in their ability to manage emotional responses effectively.

Probably more around recognising what’s happening with me. So, like helping stop me, I like catch myself in those moments as things start to escalate. Just being able to recognise those moments, and although I am not able to stop at 100% of the time, I’d say it’s probably you know at least 85–90% of the time where I am able to like stop it, reduce it. (Participant 4)

Through the skills learned in MI-DBT, mothers developed the ability to pause, reflect, and create space between their emotions and their responses. This shift from reactive to more deliberate responses was an important aspect of their growing self-awareness. The ability to mindfully recognise escalating emotions allowed mothers to choose healthier responses instead of defaulting to automatic reactions.

Just taking the time I guess to, sort of calm down a bit before I respond instead of just reacting immediately (Participant 7).

Mothers also described an increased understanding and acceptance of their disorder, which reduced internal struggles and made it easier to be mindful of their emotional state. This awareness allowed them to catch themselves in moments of emotional escalation and consciously choose more appropriate responses and behaviours.

Sometimes I’ll, once you know, if I’m sort of in a situation of, I’ll stop back, take a step back before responding in some situations, and just sort of work through my response, because obviously you know, when you’re borderline you sort of, respond you know, a little bit differently (Participant 2).

It comes down to acceptance because then I don’t fight with myself over my illness and then I’m able to be more aware of my emotional state when I react so I don’t react straight away (Participant 6).

This increased self-awareness and ability to respond more thoughtfully when emotionally aroused, allowed mothers to reflect on the broader value of emotional regulation skills, highlighting the importance of teaching these life skills to everyone.

I think it should be taught in school, those things Yeah, they’re life skills that everyone could use. Like, emotions, they don’t really get taught how to handle our emotions from a young age (Participant 1).

Furthermore, mothers described how these skills, which they practices over several years, became ingrained in their daily lives. The automaticity with which they now recognised and responded to their emotions reduced the emotional and cognitive load. This sense of mastery over their responses allowed them to feel more competent and confident in managing their day-to-day emotional experiences.

In the past there would have been conscious efforts to implement them, whereas now I think they some of those skills have just sort of become a part of how I deal with things…they’re more unconscious thoughts that have just become a part of everyday parenting (Participant 4).

I find that I don’t really have to use the skills, or I mean I probably do use them but I don’t have to actually think about using them anymore (Participant 5).

### Negotiating relationships

This theme relates to mothers’ improved confidence and ability assert themselves, set appropriate boundaries, and communicate more effectively in adult relationships. Their enhanced ability to manage conflict and interpersonal interactions was closely linked to their improved capacity to manage emotionally charged situations. Mothers shared that they felt better able to handle conflict in a calm and measured way, recognising when situations might escalate, and applying skills to prevent them fromgetting out of control.

It certainly helped me recognise the importance to trying to just stay calm and patient and you know be that adult be that wise person to just get through the situation…I learnt within my body the signs my body’s giving and my brain and all that sort of stuff (Participant 4).

I think I’m a bit more relaxed about it now…I don’t, go looking for conflict if I can help it” (Participant 2).

As they learned how to manage conflict more effectively, mothers found that MI-DBT also helped them navigate other interpersonal interactions. One mother discussed how she focused more on the broader picture in relationships rather than getting caught up in minor issues.

I think there was definitely a lot of skills that have helped me respond more effectively within relationships and things like that, or just trying to focus on the big picture instead of every little thing, but it’s hard to let go of at times (Participant 7).

Mothers described an increased awareness of their personal boundaries and respectful interactions, and opportunities to practice through MI-DBT, helped mothers influence partner relationships, friendships and work. For example, one mother described how her increased understanding of the need for self-care allowed her to negotiate with her employer to prioritise her mental health.

With my work for example I just don’t work Wednesdays at all…I can’t work the Wednesday because what what I was doing on Wednesdays for myself. That was really the biggest thing for me that I just said I for my mental health I can’t stop. Everything else I could sort of work around but that was the one thing that I just said no I I can’t do it. It’s too important for me. And the DBT sort of helped give me that confidence to recognise not not just recognise but then be able to speak up in such a way that they understood and they understood the importance of it without it coming across you know negatively (Participant 4).

Mothers also described an increased understanding of what constitutes a healthy relationship, and recognised that they deserved to be treated with respect in their intimate relationships. Many mothers shared how MI-DBT empowered them to leave relationships that were characterised by control, abuse and power imbalances.

So, it just helped me to like I, I havent really like had any lows or big lows since I left my husband. Then it’s just helped me to be able to pull myself out of those things now because I have, like things in place that I know what to do…Yeah, but it was a relief, because I didn’t realise how damaging that relationship was. Well, there was a lot of control and I guess him a bit of a narcissist…but I had to get out of there ‘cause I got hit twice, and I just never thought I’d be in that situation (Participant 1).

Overall, in time as I got better and better at dealing with things and things got worse and worse at home kind of led to me going to enough is enough with yeah, his Dad and actually kind of building my life up from there (Participant 7).

Mothers who left their partners reflected not only on the challenges of separation and negotiating care arrangements for their children, but also on the benefits of their decision. For example, the majority of those who reported leaving their partners, shared that over time, their relationship with their ex-partners improved to the point where they could communicate more effectively and even co-parent together.

we get along fine now, but we have our moments where we don’t agree with things, but most of the time we just talk in person and we understand each other a lot more (Participant 1).

Ah it changed a lot in good way. We actually separated for half a year…my husband went to see a counsellor to manage his anger problem and he got much calmer, so it’s changed my yeah feeling about him (Participant 8).

But it’s actually quite good now. We’re communicating really well now…I wrote up a parenting plan, we went through it together…it means that we are both on the same page and that we need to communicate with each other (Participant 2).

Mothers who had separated from their partners also noted that their ex-partners became more involved in their children’s lives after the separation, contributing to more balanced caregiving responsibilities.

like their Dad never did a lot with us, so it was always me who took them on outings and stuff…Since we’ve split, like they have the weekends with their Dad I guess that’s made it better like they see him and get to do things with him (Participant 1).

These life-long skills in negotiating interpersonal interactions learned through MI-DBT also extended to new relationships. Mothers who had entered new partnerships after completing the program reported healthier interactions compared to their previous relationships While mothers acknowledged that conflicts sometimes arose, they felt better equipped to manage these situations.

Yeah, we’re still working on that. He doesn’t really do conflict at all so, that’s something we’re yeah sort of working on because, yeah whenever I try to, discuss things it, yeah, he doesn’t really talk so, that’s something yeah. Eventually we do but it just takes a lot of pressing and, yeah (Participant 2).

### We want more!

This theme reflects the mothers’ desire for more targeted and specific support following the completion of the MI-DBT intervention. Whether co-parenting, parenting alone, or with a new partner, mothers described the overwhelming burden of parenting falling primarily on them. This sense of isolation, while not unique to mothers with BPD [36], added to the stress of balancing motherhood with managing a mental illness. Many mothers shared that they had limited support, with partners working long hours, being physically or emotionally unavailable, and friends and family often not present to help. Some mothers also expressed feeling let down and abandoned at critical moments, such as during pregnancy, which left them struggling to manage both their mental health and parenting responsibilities. They expressed that having more support would make these challenges easier to navigate.

their Dad doesn’t get home until 5 most nights if a bit later, and he’s gone at 6.30, 7 in the morning so, it is literally me 80% of the time and then he goes away at times…like there’s literally like no breaks at all…So then I, it is my responsibility to do it all (Participant 3).

I don’t have like family support so it makes it, makes it hard (Participant 6).

Despite the usefulness of the skills learned in MI-DBT, mothers expressed variation in their ability to apply these due to the practical demands of parenting. Almost unanimously, mothers shared that the physical and emotional toll of parenting left them with limited time, energy and space to fully utilise the skills they had learned.

Sometimes it’s just not being able to physically separate when you know that’s what you really need in that moment, that’s probably been the biggest barrier (Participant 4).

As such, despite many mothers receiving psychological support services, all of the mothers expressed a strong desire for a refresher of the skills they had learned. Although they felt more confident in their parenting and their ability to manage stressful situations, they also shared feelings of uncertainty and occasional inadequacy. They acknowledged the need for flexibility in order to adapt to the changing needs of their children as they grew older, and expressed that it would be helpful to revisit and practice the skills in this context.

But even just how to learn now as they’re getting a bit older, how to manage their well-being with their mental health stuff, my like my eldest now being 7, she can be quite full on at times and things like this so like I’m there going I wonder if there’s something that now that they’re older that we could try and stuff. (Participant 3)

I feel like if there was that refresher course or just something, to follow on, you know obviously, like even just the once it it’s been what a good 3 years since we’ve done it and stuff like that, and obviously I know for me I’ve had more kids (Participant 3).

Mothers suggested that having a dedicated group to attend physically would help them commit to practicing the skills. They believed that having a communal space, rather than trying to manage things in isolation at home, would enable them to prioritise their self-care and skill practice. This desire for mutual support and solidarity was expressed by several mothers, who felt that their parenting burden was often solely on their shoulders.

if there was an updated not an updated, but an up, another sort of short you know, a few weeks or whatever, once a week or whatever to go somewhere and do it. It’s probably a lot easier than just, trying to do it at home and things like that. Because even though I’ve got the folder, just in the bedroom and things like that, it is a lot harder just to pull everything out with the kids around and really focus on it (Participant 3).

I’m on the waiting list to do that again, just to revise the skills. (Participant 6)

Mothers also voiced that the psychological support they received after MI-DBT, while helpful, often did not meet their specific needs as mothers with BPD. They expressed feeling a gap in post-partum support that catered to their unique circumstances.

so, I think it’s a really, really good program but I guess like maybe some sort of refresher or follow-up down the line like wouldn’t hurt…I think it’d be good to yeah refresh a lot of things, like it’s hard to find psychologists who practice like DBT as their, like method (Participant 7).

Many mothers had engaged with ‘generic’ DBT prior to (and to a lesser extent, after) engagement with MI-DBT. While they found it useful for learning basic skills, they emphasised that MI-DBT’s parenting focus was especially valuable in meeting their needs. Regardless of when they completed DBT, mothers appreciated having the opportunity to consolidate skills across both approaches.

The … [name of group removed for anonymity] one really helped me focus in on being a parent. Whereas the normal standard one just didn’t have those associations between okay but I’m a Mum, I’ve got a baby (Participant 4).

Despite ongoing challenges, mothers overwhelmingly reported that, although parenting remained difficult, their emotional reactions were less intense and frequent, and they felt better equipped to handle the everyday demands of parenting.

Like things do still feel hard I guess all of the time, because it’s you know work as a single Mum with a kid with special needs, and they’re still very young, but I don’t feel like doomed and hopeless like I used to (Participant 7).

## Discussion

Given the limited availability of services for mothers with BPD, and the paucity of research on long-term intervention impacts, this study investigated the experiences of mothers 3 years post-Mother-Infant Dialectical Behaviour Therapy (MI-DBT), a parenting-specific DBT intervention. A thematic analysis of experiences revealed the complexities of parenting with BPD, highlighting four key themes: the role of MI-DBT in keeping children in mind, enhanced reflective capacity and awareness of self, improved relational negotiation skills, and the continued need for support post-intervention.

Overwhelmingly, mothers reported improvements in emotional regulation, distress tolerance, and mindfulness skills, which allowed them to better manage their emotions and respond more thoughtfully to their children. Furthermore, increased understanding and awareness of their children and their needs, increased empathy, and a desire to learn more about their children, resulted in an improved ability to keep their children in mind. While many mothers attributed these improvements to the skills learned in MI-DBT, it is important to consider that other therapeutic or personal experiences over the past three years may have also played a role. Previous research has shown that mothers’ reflective ability can be compromised when emotionally aroused [16, 71, 72], yet participants in this study articulated numerous accounts of keeping their children in mind during affective intensity, confirming the improvement noted at earlier follow-up [58]. While these accounts suggest improvement, we cannot definitively attribute these changes directly to MI-DBT without further exploration of causal links.

Mothers in this study also reported strong identities as parents, and a deep sense of responsibility to do the best they can for their children. These findings resonate with previous literature [15, 73] where mothers reflected on feelings of guilt and shame about their behaviour and its potential impact on their children. While guilt remained a source of distress, mothers in this study also described it as a motivator for behaviour change, which aligns with other research on parenting with BPD [14]. Interestingly, while many mothers attributed improvements in their self-awareness to MI-DBT, guilt was also described as a key motivator for behaviour change, suggesting that a combination of program-related skills and personal reflections contributed to this shift.

Echoing previous findings [14, 15] mothers with BPD described parenting as intensely stressful compared to mothers without the diagnosis [36]. However, transcripts from this study also revealed that some mothers expressed a sense of joy and reward in parenting, which contrasts with earlier findings [16, 36]. This may indicate a perceived improvement in parenting competence from the program. It is possible that the mothers’ perceived increases in parenting competence and their enhanced ability to manage their own emotions buffered some of the typical parenting stress. However, it remains important to consider that these improvements may have developed as part of a broader therapeutic journey, with MI-DBT serving as one contributing factor. While emotional instability is a hallmark feature of BPD [18], mothers reported increased competence in their ability to manage their own emotions in intense moments and be able to respond appropriately to their children, which they attributed to learning through MI-DBT. This finding contrasts with previous literature suggesting that mothers with BPD struggle to contain their own emotions and respond sensitively to their children [16]. Similarly, mothers reported an increased understanding and acceptance of their disorder, accompanied by a level of self-compassion when things did not go as planned. While continuing to feel guilt regarding their previous behaviour and the impact on their children, their narratives were also interspersed with acceptance and non-judgement. Many reflected on previous deficits in emotional regulation and expressed pride in the changes made, and confidence in trying something different.

Despite feeling more confident in managing their emotions and responding more sensitively to their children, many mothers continued to doubt their parenting efficacy. This aligns with previous qualitative literature, where mothers with BPD reflect on the self-doubt, lack of confidence, guilt and worry about their parenting [14, 15]. This further highlights mothers’ sense of responsibility towards their children and the importance of their role as mothers.

Mothers in this study also described feeling that the parenting burden was solely on them, echoed by narratives of compromised partner relationships. These responses are similar to those in Dunn and colleagues’ [16] research, in which participants felt overwhelmingly unsupported in their parenting role. Although mothers reported healthier relationships with partners post MI-DBT, these narratives lacked reference to partners as supportive figures, highlighting the continued struggle with compromised social networks common in BPD [73], further adding to the stress associated with parenting.

A noteworthy finding was the reported efficacy of MI-DBT in helping mothers navigate challenging relationships. Participants reported noticeable improvements in managing interpersonal interactions, often linking these changes to skills learned in MI-DBT, including enhanced assertiveness, boundary-setting, conflict management and communication skills. However, it is important to acknowledge that these changes could also be influenced by other therapeutic experiences or personal growth over the three-year period. Given that individuals with BPD often face chaotic and unstable relationships [18], it is perhaps unsurprising that the mothers in this study reported previous relationships dominated by power and control dynamics. However, post-MI-DBT, mothers were keen to highlight their increased understanding of what constitutes a healthy relationship, as well as their newfound proficiency in setting boundaries with others. Consequently, they reported having healthier interactions with those around them, encompassing both previous and current partnerships. Considering the high prevalence of abuse experienced by mothers with BPD within their relationships [74], interventions such as MI-DBT where mothers articulate improvements in their intimate relationships pose significant benefits for all involved.

Moreover, the data reveals an empowering shift in awareness among these mothers. Some mothers reported reaching a critical realisation that they were deserving of better treatment, leading them to make the decisive step to part ways with their partners. This finding, while somewhat unexpected, underscores the profound transformative potential of programs such as MI-DBT. This capacity for fostering deep, holistic change underscores the potency of MI-DBT and adds a compelling layer to our understanding of its impacts.

While mothers described the skills learned in MI-DBT as useful, they continued to feel isolated and lacking in support from their social and professional networks. Similar to previous research, mothers felt unsupported by services, characterised by lack of access, limited support availability, and wanting something specific to their needs as parents [16]. Many mothers in the current study described lengthy waits to access either MI-DBT or traditional DBT interventions, despite an almost unanimous desire for further support. Mothers also shared the value of connecting with other mothers within the MI-DBT program, suggesting that refresher or follow-up sessions could support maintaining these networks, and reduce the reported isolation experienced.

This highlights both the need for opportunities for connections with others, and improved access to and availability of services for this population.

## Strengths

Actively seeking out participant responses in a non-judgmental setting allowed mothers with BPD to provide candid insights into their experiences post-MI-DBT.

Additionally, most participants had previously been admitted to a mother-baby inpatient unit in South Australia, suggesting a sample with considerable mental health challenges. This makes the reported changes particularly noteworthy, suggesting the potential for lasting improvements and the possibility of a positive future.

## Limitations

Although the purpose of this study was to understand participants’ experiences of life after MI-DBT, exploring the impacts of the intervention was also an aim. As such, interview questions and interviewer follow-up may have limited or impacted participant responses, thereby not gaining as full an understanding of life after MI-DBT. For example, although mothers indicated improvements and the ability to keep their children in mind, descriptions of how mothers support their children’s emotional regulation were limited, as were specifics relating to the mother-child relationship. Interview questions did not deeply explore how mothers applied skills to support their children’s emotional regulation post-MI-DBT. Future studies could target this area.

Similarly, interviews were conducted by different individuals, which resulted in variations in the length and depth of participants’ responses. In cases where more detailed information was needed, conducting a follow-up interview could have provided additional depth. Additionally, having a different individual code the interviews, separate from those who conducted them, is a limitation. This separation could have led to potential misinterpretations or a lack of nuanced understanding of the context in which the responses were given.

Furthermore, while identified changes were made by these mothers, it is impossible to conclude that these changes were directly a result of participation in MI-DBT. Though mothers credited MI-DBT for many of their improvements, some had received other forms of therapy, making it difficult to isolate the effects of MI-DBT alone. Finally, while 29 women were eligible for follow-up, only eight women responded to recruitment efforts and participated in interviews. It is not known why those who did not participate did not respond. It may have been that the women who participated had motivations to be interviewed (for example a particularly positive or negative experience) or felt more able to do so (for example, having experienced improvements in their mental health). More interviews are therefore needed as more women become eligible for three-year follow-up.

## Future directions

Given the high rates of neurodivergence in the offspring reported by mothers in this study, future research could explore the intersection of MI-DBT with parenting neurodivergent children. Additionally, considering the identified limitations of the study, future research might also include a control group or a modified protocol that includes both MI-DBT and parent-infant therapy to better evaluate the specific impacts of each intervention.

This research highlights the benefits of MI-DBT and interventions to help both mothers and children, and highlights the need for continued support and service provision after the completion of MI-DBT. Program improvements could consider regular follow-ups, reunions, or a short group one year after MI-DBT completion. This would respond to participants’ reported struggle with flexibly responding to the changing developmental needs of their children.

## Conclusion

In conclusion, the current study provides further insight into mothers’ experiences of life after MI-DBT, and the impact the program has had on their lives. Mothers expressed improvements in their ability to keep their children in mind, respond differently when in emotional situations, and manage interpersonal interactions within adult relationships. These changes can have significant implications for the trajectory of mothers and their children. While MI-DBT provided participants with essential skills in emotional regulation, interpersonal relationships, and parenting, this study highlights that the program is only one part of a longer-term process of managing BPD. Parenting remains difficult even in the best of circumstances, and BPD’s ongoing nature means that these mothers may continue to face challenges. The findings suggest that continued support and broader social services are critical for sustaining these improvements over time. This research is the first to explore the longer-term experiences of mothers with BPD following an intervention specifically targeted to them as mothers, and in giving voice to this vulnerable group of women, this research has deepened our understanding of the complex challenges experienced as part of parenting with BPD.

## Electronic supplementary material

Below is the link to the electronic supplementary material.

Supplementary Material 1

Supplementary Material 2

Supplementary Material 3

## Data Availability

The data that support the findings of this study are available from the Women’s and Children’s Health Network, but restrictions apply to the availability of these data, which were used under license for the current study, and so are not publicly available. However, the data are available from the authors upon reasonable request and with the permission of the Women’s and Children’s Health Network.

## Abbreviations

BPD: Borderline personality disorder
DBT: Dialectical Behaviour Therapy
MI-DBT: Mother-Infant Dialectical Behaviour Therapy
WCHN: Women’s and Children’s Health Network
WCHN HREC: Women’s and Children’s Health Network Human Research Ethics Committee

## Footnotes

1 To date, 21 groups have been run at a mother-baby inpatient unit in South Australia, and multiple Children’s Centres across metropolitan Adelaide since November 2013. 142 women have completed some, however in some cases not all, of the research components of the program (e.g., incomplete quantitative data). Participants initially consisted of previous inpatients at a mother-baby inpatient unit in South Australia, and later included those from external referrers.

2 Participant demographics were not provided due to hospital privacy guidelines.
