# Supplementary material for: A thematic analysis of the subjective experiences of mothers with borderline personality disorder who completed Mother-Infant Dialectical Behaviour Therapy: a 3-year follow-up
Source: Borderline Personal Disord Emot Dysregul. 2024 Oct 28;11:25. doi: 10.1186/s40479-024-00269-w (PMC11514748; doi:10.1186/s40479-024-00269-w)
Supplement: Supplementary file 1 — Supplementary Material 1 [file 40479_2024_269_MOESM1_ESM.docx]

**Appendix A – Intervention Protocol**

MI-DBT is a structured manualised program that involves a weekly 2.5-hour group therapy session over 24 weeks. The sessions teach mothers DBT skills and how to apply these to parenting, where participants learn skills to manage parenting stress, develop secure infant attachments and support their infant’s emotional regulation. The sessions are divided to cover four DBT skills:

*Mindfulness*

Using concepts of Acceptance and Commitment Therapy, Mindfulness provides participants with the skills to be more focused on the present moment, and how to manage difficult thoughts and emotions in line with their parenting values.

*Distress Tolerance*

Distress Tolerance skills provide strategies to be better able to manage difficult emotions and situations. Using concepts from the Circle of Security, participants learn to recognise their emotional triggers and how to support the infant in learning distress tolerance skills.

*Emotional Regulation*

Emotional Regulation skills provides participants with a better understanding of, and strategies to manage emotions for themselves and their infant. Mothers are taught how to support their child’s emotional regulation while distressed themselves.

*Interpersonal Effectiveness*

Interpersonal Effectiveness skills assists participants to negotiate relationships, such as through boundary-setting, assertiveness, problem-solving and negotiation skills, in respectful and healthy ways. Participants also learn how to support their child’s social development.

While mothers participate in the formal DBT skills session (2.5-hour), their infants are cared for by paid professionals or volunteer carers. After the sessions, mothers are reunited with their infants in a 15-minute structured activity using song and dance, allowing the opportunity for mothers to practice the new skills they have just learnt.
