# Supplementary material for: A thematic analysis of the subjective experiences of mothers with borderline personality disorder who completed Mother-Infant Dialectical Behaviour Therapy: a 3-year follow-up
Source: Borderline Personal Disord Emot Dysregul. 2024 Oct 28;11:25. doi: 10.1186/s40479-024-00269-w (PMC11514748; doi:10.1186/s40479-024-00269-w)
Supplement: Supplementary file 2 — Supplementary Material 2 [file 40479_2024_269_MOESM2_ESM.docx]

**Appendix B –Interview Schedule**

**Post-group interview questions**

Last time you were interviewed, we talked about what led you to enrol in MI-DBT, some of the struggles you were experiencing, and your hopes for the program and your future. How have things been going for you since you started the MI-DBT journey?

How have you been feeling lately?

Before starting MI-DBT, you talked about [particular issues]. How are you feeling about that now?

- Repeat as needed, as per discussions from pre-group interview

You mentioned [behaviour] last time we spoke. Are you still finding this is happening, or has there been a change?

- Prompt to elaborate as needed

Tell me about (infant/child)

- Prompt for infant personality/personhood
- Prompt for behaviour, age milestones

What’s your relationship with (infant) like?

- What’s the best part about parenting (infant)?
- What are some of the challenges you face with (infant)?
- Are there particular times that you find more challenging (e.g. sleep, feeding etc)
- What do you find helpful when (challenge) happens?
- What do you do for yourself when that happens?
- What do you do for (infant)?

Have you noticed a change in your relationship with [infant] since starting MI-DBT?

Before MI-DBT you mentioned [specific challenge with infant]. How are you feeling about that now?

How are you feeling about your relationships at the moment?

- Prompt for partner
- Prompt for family
- Prompt for friends

Have you noticed any changes in the way you respond to difficulties since starting MI-DBT?

- Prompt for relationship with infant
- Prompt for relationship with partner/family/friends

Overall, how was your experience of MI-DBT?

- Prompts for any suggestions for improvement

Are you connected with any other mental health services at the moment?

- Prompt for community services, private psychology/psychiatry/counselling

What has it been like to attend the MI-DBT group at [children’s centre]?

Did the group being run at [children’s centre] influence your decision to attend?

- Prompt for positives and negatives of children’s centre (location, atmosphere, resources etc)

Wrap up – is there anything else you’d like me to know about your journey so far?
